# CARRoT: R-package for predictive modelling by means of regression, adjusted for multiple regularisation methods

**DOI:** 10.1371/journal.pone.0292597

**Published:** 2023-10-12

**Authors:** Alina Bazarova, Marko Raseta

**Affiliations:** 1 Jülich Supercomputing Center, Forschungszentrum Jülich, Jülich, Germany; 2 Helmholtz AI, Munich, Germany; 3 Department of Molecular Genetics, Erasmus MC, Rotterdam, Netherlands; Dhaka University, BANGLADESH

## Abstract

We present an R-package for predictive modelling, *CARRoT* (*C*ross-validation, *A*ccuracy, *R*egression, *R*ule *o*f *T*en). *CARRoT* is a tool for initial exploratory analysis of the data, which performs exhaustive search for a regression model yielding the best predictive power with heuristic ‘rules of thumb’ and expert knowledge as regularization parameters. It uses multiple hold-outs in order to internally validate the model. The package allows to take into account multiple factors such as collinearity of the predictors, event per variable rules (EPVs) and R-squared statistics during the model selection. In addition, other constraints, such as forcing specific terms and restricting complexity of the predictive models can be used. The package allows taking pairwise and three-way interactions between variables into account as well. These candidate models are then ranked by predictive power, which is assessed via multiple hold-out procedures and can be parallelised in order to reduce the computational time. Models which exhibited the highest average predictive power over all hold-outs are returned. This is quantified as absolute and relative error in case of continuous outcomes, accuracy and AUROC values in case of categorical outcomes. In this paper we briefly present statistical framework of the package and discuss the complexity of the underlying algorithm. Moreover, using *CARRoT* and a number of datasets available in R we provide comparison of different model selection techniques: based on EPVs alone, on EPVs and R-squared statistics, on lasso regression, on including only statistically significant predictors and on stepwise forward selection technique.

## Introduction

Linear regression remains a first-choice tool for building predictive models in a vast variety of applied problems. Interpretability and simplicity of implementation often make it into a ‘gold standard’ to compare with when developing more complex models for prediction. In the assessment of medical datasets, this approach becomes particularly important as the comprehensive risk scores for patient stratification can be easily derived from the regression coefficients and are therefore accepted and well-understood by practitioners. Moreover, given that every infinitely differentiable function can be approximated by a Taylor polynomial, including higher order interactions terms between independent variables can help unravel potential non-linear relationship between those and the outcome variable.

Often in the studies of a very specific medical condition, it is the case that feasible sample size is rather moderate while the number of explanatory variables is high [1, 2], therefore including too many of them into the predictive model may lead to overfitting. Events per Variable (EPV) rules are a common tool in Medical Statistics used to avoid the latter, initially introduced as ‘one in ten rule’ [3] and verified via extensive simulations. Since then, albeit substantial criticism, both on the number of events [4–7] and on the general concept of the EPV, [8, 9], the bespoke rule remains widely used and highly cited.

The latter two papers address such problems as handling separated datasets and biased regression coefficients. Furthermore, the authors assess robustness and performance of multiple different models in the context of EPV rules via a number of simulation studies. In conclusion they suggest targeting certain values of out-of-sample mean absolute prediction error and mean squared prediction error to determine the sample size. Of note is that the above two papers do not aim to study prediction problems in small sample sizes. Moreover, although there is a heterogeneity between performance of different models in cases EPV rule is set to low values, it seems to significantly decrease as the latter grows. In turn, the impact of the EPV rule on the predictive power decreases with the fraction of events across a number of different regression models presented in the manuscripts. This observation is especially important for the studies with low prevalence outcomes.

In [10, 11] authors provide detailed step-by-step instructions of estimating sample size for fitting binary and continuous outcomes. These are mainly based on *R*^2^-statistics assessing goodness of fit and global shrinkage factors aiming to reduce overfitting. However, the authors also point readers to certain examples where low *R*^2^ values do not imply poor predictive performance of the model.

In this paper, rather than estimating optimal sample size in order to provide a robust model fit, we concentrate on a somewhat inverse problem. Namely, given a dataset of fixed sample size and a certain number of predictive variables determine a model leading to the best predictive performance. The latter, along with the model robustness, is assessed by means of multiple internal validations, essential for model development [12, 13].

*CARRoT* is implemented as a package within statistical software R [14]. It performs exhaustive search over all subsets of explanatory variables, also known as ‘best subset regression’ [15] subject to custom constraints. These are firstly the ones translating available sample size into the properties of feasible models. Namely, user can specify a certain EPV rule (with the default ‘one in ten’, EPV = 10) to be satisfied, or alternatively verify, whether the criteria described in [10, 11] are met for each model. These two methods can be applied both separately and in combination. In section “Results and discussion”, we provide examples run on publicly available datasets, illustrating the usage of each one of them.

Secondly, *CARRoT* provides the option of expert knowledge integration, thereby introducing further constraints into the process of model selection. In other words, *CARRoT* is adaptive as its prediction algorithm can be tailored to specific situations. This arises in practice when there is an expert consensus on which variables will necessarily be a part of predictive model, and the scientific question to be answered is whether adding a number of new independent variables results in increased predictive power. Another common situation occurs during a General Practitioner appointment when a number of measurements have to be taken, and decisions made on the spot. Indeed, as the study [16] demonstrates, length of the GP appointment does not exceed 5 minutes for more than 50% of world population. Consequently, this practical temporal constraint naturally gives rise to an upper bound on the number of explanatory variables the model can comprise. Moreover, *CARRoT* also allows one to reduce the search space by focusing only on those models where correlation between the explanatory variables is bounded by a specified constant.

To ensure internal validation, *CARRoT* performs hold-out procedure a specified number of times by randomly splitting the input dataset into the training and test sets. For each training set, the package fits regression models based on all possible subsets of variables subject to constraints, and then assesses their predictive power on the corresponding test set. The average predictive power for each model is then computed over all the test sets, and the variables constituting the models with the highest average predictive power are then returned.

There are a number of R packages which perform an exhaustive search over all possible subsets of variables, and then output the ‘best’ one, based on a certain type of statistics. These are e.g. *glmulti* [17], *bestglm* [18], *BeSS* [19], *meifly* [20], *lmSubsets* [21], *subselect* [22], *kofnGA* and [23] to name just a few. However, such packages, unlike *CARRoT*, lack a built-in cross-validation procedure and therefore do not work directly with predictive power. Furthermore, although the option of restricting the number of variables is usually available in the existing packages, it is not equivalent to the EPV constraint when categorical variables are present in the dataset. In such packages *R*^2^ statistics is frequently used to rank the models, however this is not enough to ensure that the model is feasible for the given sample size either. Therefore, to the best of our knowledge, this is the first package that allows one to restrict the model selection based on sample size for regression criteria within a hold-out framework.

## Methods

### EPV rule

EPV rules were originally introduced as an overfitting reduction tool, however, this procedure naturally gives rise to the upper bound on the number of variables to be included in the model. Let *X*_1_, …, *X*_*n*_ be the dependent variables, *Y* be an independent variable, and *g* be a link function corresponding to the type of the outcome. Then, let

$$\begin{array}{c} g(Y)=\sum_{k=1}^{p+q}\beta_{i_{k}}X_{i_{k}},\;\left\{i_{1}\ldots i_{p+q}\right\}\subset \left\{1\ldots n\right\} \end{array}$$ (1)

where $X_{i_{1}},\ldots ,X_{i_{p}}$ are continuous and $X_{i_{p+1}},\ldots ,X_{i_{p+q}}$ are discrete categorical variables, each with $l_{i_{j}}$, *j* ∈ {*p* + 1…*p* + *q*} numbers of categories. Assume that the desired EPV rule is the rule of *r*, where *r* is often chosen to be 10. This means that depending on the type of the outcome (see Table 1, last column), in order to include *m* variables into a regression model, sample size/number of events in the least likely category has to be at least *r* times *m*.

**Table 1:** Link function. The table of link functions corresponding to different types of outcomes.

Distribution	Support	Type	Link function	EPV rule applied to
Normal	(−∞, + ∞)	Linear-response data	Identity	whole sample
Bernoulli/Categorical	{0, …, *k* − 1}	Outcome of single *k*-way occurrence	$\text{ln}\;(\frac{Y}{1-Y})$	least likely category

The last column prescribes how Events in the EPV rules should be counted

Speaking more rigorously, the model (1) is feasible if the following is satisfied

$$\begin{array}{c} r(p+\sum_{j=p+1}^{p+q}\left(l_{i_{j}}-1\right))\leq N \end{array}$$ (2)

where *N* is either the given sample size for continuous outcomes or the number of events in the least likely category for categorical ones. Note that categorical variables with 5 or more numerical categories can be treated as continuous [24]. The default value for *r* is 10 which corresponds to ‘one in ten’ rule by [3].

### Predictive power and hold-out procedures

During each hold-out the dataset of size *N* is split into the test and training sets of size ⌊*Nα*⌋ and *N* − ⌊*Nα*⌋ respectively, where *α* is a user defined parameter (with 0.9 as the default value) corresponding to the percentage of the sample to be used for training. For each such partition, we fit all feasible regression models to the training set and assess their predictive power on the corresponding test set.

Let S stand for the set of all feasible subsets of predictors for a given hold-out, ${\left\{Y_{j_{i}}\right\}}_{i=1}^{\left\lfloor N\alpha \right\rfloor }$ for the values of outcome on the test set and ${\left\{{\hat{Y}}_{j_{i}}^{S}\right\}}_{i=1}^{\left\lfloor N\alpha \right\rfloor }$ for their estimates based on the linear regression model corresponding to the subset of predictors S∈S. The corresponding vectors of absolute and relative errors will respectively read

$$E_{a}^{S}={\left(Y_{j_{1}}-{\hat{Y}}_{j_{1}}^{S},\ldots ,Y_{j_{\left\lfloor N\alpha \right\rfloor }}-{\hat{Y}}_{j_{\left\lfloor N\alpha \right\rfloor }}^{S}\right)}^{T},E_{r}^{S}={\left(\left(Y_{j_{1}}-{\hat{Y}}_{j_{1}}^{S}\right)/Y_{j_{1}},\ldots ,\left(Y_{j_{\left\lfloor N\alpha \right\rfloor }}-{\hat{Y}}_{j_{\left\lfloor N\alpha \right\rfloor }}^{S}\right)/Y_{j_{\left\lfloor N\alpha \right\rfloor }}\right)}^{T}$$

We define absolute and relative errors for the model corresponding to subset *S* as $\left|\right|E_{z}^{S}{\left|\right|}_{1}/\left\lfloor N\alpha \right\rfloor$, where ||⋅||_1_ is an *L*_1_-norm and *z* ∈ {*a*, *r*} respectively.

For continuous variable prediction each linear regression model S∈S is ranked by relative and absolute errors averaged over the performed hold-outs. Models *S*_*a*_ and *S*_*r*_, minimizing absolute and relative errors respectively, are then reported.

For categorical variable predictions, the output of the multinomial regression are the odds ratios transformed into probabilities and consequently into categories as follows

$$\begin{array}{c} P\left({\hat{Y}}_{i}^{S}=k\right)=\frac{\text{exp}\;({\hat{P}}_{ik})}{1+\sum_{l}\;\text{exp}\;({\hat{P}}_{lk})},\;k=\left\{0,\ldots ,L-2\right\}, \end{array}$$ (3)

$$\begin{array}{c} {\hat{Y}}_{i}^{S}=\text{arg}\;\underset{k}{\text{max}}\left\{P\left({\hat{Y}}_{i}^{S}=k\right)\right\} \end{array}$$ (4)

where ${\hat{P}}_{ij}$ stands for the odds ratio of the *j*th category against the reference one and *L* is the number of outcome categories. The reference category is always the one with the highest value. If the outcome is non-numeric, it is converted into numeric one in such a way that the least frequent category has the highest value and, hence, is the reference category.

Predictive power of the models for categorical dependent variables is quantified using accuracy. To be more specific, the accuracy vector for a model S∈S is defined by

$$\begin{array}{c} A^{S}=\left(I\{\right|{\hat{Y}}_{j_{1}}^{S}-Y_{j_{1}}\left|=0\},\ldots ,I\{\right|{\hat{Y}}_{j_{\left\lfloor N\alpha \right\rfloor }}^{S}-Y_{j_{\left\lfloor N\alpha \right\rfloor }}{\left|=0\}\right)}^{T}, \end{array}$$ (5)

where *I* is the indicator function of the event. Average accuracy is defined analogously to the linear case.

Predictive power of the models for binary outcomes can also be quantified by computing AUROC values, defined in a standard fashion, with its average value derived exactly as before.

Each hold-out procedure yields |S| values of predictive power corresponding to every feasible subset of predictors. The number |S| may vary in case of categorical outcomes, since two training sets belonging to different partitions do not necessarily contain the same number of least likely outcomes. This means that predictive models evaluated during one procedure may not be evaluated during the other. If the model did not appear in all hold-outs, its average predictive power will still be calculated and scaled appropriately. In practice, there are situations when, due to a very rare type of dataset partition into training and test set, a certain model has high predictive power simply because it was not cross-validated sufficient number of times. This can be resolved by setting an upper bound on the number or weight of the predictors in a model, as it will eliminate those extremely unlikely situations when such models are feasible.

### Additional sample size criteria

The authors of [10, 11] propose to estimate sample size required to fit a particular regression model based on multiple quantities. These are the global shrinkage factor, difference between apparent and adjusted *R*^2^ statistics, the residual standard deviation estimate and the mean predicted outcome estimate for the continuous case. For binary case, instead of standard deviation and mean, an estimation of overall risk in the population are used. Shrinkage factor and difference between apparent and adjusted *R*^2^ statistics are the two measures to evaluate the problem of overfitting on the relative and absolute scale respectively. Required sample size ensures that the first one is close to 1 whilst the second one is close to 0 (> 0.9 and < 0.05 respectively as the authors recommend). The remaining criteria guarantee an adequate estimation of the basic parameters from the population.

When calculating appropriate sample size for a regression model according to [10, 11], one also needs to take into account the anticipated values of *R*^2^-statistic, mean outcome and the population variance or alternatively an outcome proportion. This typically requires either using the data from previous studies or an educated guess, and can therefore be rather demanding. In *CARRoT*, the hold-out concept of splitting the data into training and test set naturally allows to decide whether a model is feasible for the target sample size based on the values computed on the training set. Namely, the above quantities are computed on the training set and, in case they satisfy the given constraints, predictive power of the model is assessed on the corresponding test set. Otherwise, it is declared infeasible for the current hold-out and the validation step is skipped.

Therefore, in case of linear regression, apparent *R*^2^ of the model on the training set has to satisfy the following condition, which follows directly from the formulae for global shrinkage factor and difference between apparent and adjusted *R*^2^-statistic provided in [10, 11]

$$\begin{array}{c} R^{2}>\text{max}\;(1-\text{exp}\;(\frac{2-p}{0.1n_{tr}}),\frac{p-0.05(n_{tr}-1-p)}{p}) \end{array}$$ (6)

where *p* is the weight of the model and *n*_*t*_*r* is the size of the training set. Similarly, for error margins of the residual variance and mean outcome we use the following formulae

$$\begin{array}{c} M_{v}=\sqrt{\text{max}\;(\frac{\chi_{1-\frac{0.05}{2},n_{tr}-p-1}^{2}}{n_{tr}-p-1},\frac{n_{tr}-p-1}{\chi_{1-\frac{0.05}{2},n_{tr}-p-1}^{2}})} \end{array}$$ (7)

$$\begin{array}{c} M_{o}=t_{1-\frac{0.05}{2},n_{tr}-p-1}\frac{\hat{\sigma }}{\sqrt{n}\hat{\alpha }}, \end{array}$$ (8)

where the notation is as in (6), *M*_*v*_, *M*_*o*_, ${\hat{\sigma }}^{2}$, $\hat{\alpha }$ are margins of error and training set estimates of residual variance and mean outcome respectively. The package allows to specify the desired margin *M*_*o*_ = *M*_*v*_ in (7) to be chosen.

For the binary case we ensure that the following conditions are satisfied

$$\begin{array}{c} S_{VH}=1-\frac{p}{-2(\text{ln}\;L_{0}-\text{ln}\;L_{m})}>0.9 \end{array}$$ (9)

$$\begin{array}{c} \frac{1-\text{exp}\;(-2\left(\text{ln}\;L_{0}-\text{ln}\;L_{m}\right)/n_{tr})}{1-\text{exp}\;(2L_{0}/n_{tr})}\left(1-S_{VH}\right)<0.05, \end{array}$$ (10)

where *S*_*VH*_ is the Van Houwelingen’s shrinkage factor, *L*_0_ and *L*_*m*_ are the log-likelihoods of the model with no predictors and the full model currently being assessed, respectively. The error margin for outcome proportion in the population can be customized and is computed as follows

$$\begin{array}{c} M_{b}=1.96\sqrt{\frac{\hat{\phi }\left(1-\hat{\phi }\right)}{n}}, \end{array}$$ (11)

where *M*_*b*_ is the error margin and $\hat{\phi }$ is the estimated outcome proportion on the training set. Note that all formulae (6)–(11) are either taken or derived from the ones in [10, 11].

### Other features and computational complexity

In case when all predictors in a given dataset are either continuous or binary the number of all subsets constrained by an EPV rule will be $\sum_{i=1}^{w}(\begin{array}{c} n\\ i \end{array})$, and therefore if *w* > *n*/2 the problem of finding all subsets becomes computationally infeasible with the increase in sample size. However, if *w* < *n*/2 and is fixed, the sum can be bounded by $O((\begin{array}{c} n\\ w \end{array}))$, and therefore complexity grows polynomially when it is the number of variables rather than the sample size that is increasing. In practice, the set of explanatory variables is often a mixture of those with different weights, allowing for more computational feasibility. Note that when working with medical datasets, e.g., creating a score, the maximal number of predictors is often bounded by 20 (e.g. due to the time constraint and necessity to take measurements on the spot, [16], therefore making the problem computationally feasible.

Additional features of the package ease the computational burden of the algorithm. In case there is a ‘fixed’ part of the predictive model (consensus between medical experts reached, meaning specified variables have to be a part of the model), the number of all feasible subsets will be reduced by $\sum_{i=w-l+1}^{w}(\begin{array}{c} n\\ i \end{array})-1$ (where *l* is the weight of the “fixed” subset). This reduction is not insignificant, as it removes the largest terms of the partial sum which takes predictors without this constraint into account. Similarly, on top of specifying minimum and maximum number of predictors, as well as the maximum weight of a model (where weight of a model equals the sum of weights of variables it comprises), *CARRoT* also allows users to eliminate models containing correlated predictors from the selection process by specifying the highest allowed correlation between the independent variables (default is set to 1).

### Higher order models

Subject to feasibility restricted by computational complexity (e.g. *w* ≪ *n*), in order to increase the predictive power, one can include second order terms, such as squares and all pairwise interactions between the variables. As previously indicated, the complexity growth is significantly slower with the number of variables when the sample size is fixed, although here the increase is still highly significant growing from *O*(*n*^*w*^) to *O*(*n*^2*w*^). Function quadr transforms the set of given numeric variables into the one containing quadratic terms in a following way:

$$\begin{array}{c} \left(a_{1},\ldots ,a_{k}\right)\to \left(a_{1},\ldots ,a_{k},a_{11},\ldots ,a_{1k},a_{22},\ldots ,a_{2k},\ldots ,a_{k-1,k-1},a_{k-1,k},a_{kk}\right), \end{array}$$ (12)

where **a**_*i*_ is the *i*th predictor (*m*-dimensional vector), **a**_*ij*_ is the variable obtained by coordinatewise multiplication of **a**_*i*_ and **a**_*j*_.

Similarly, there is also a function cub which includes cubic terms, transforming the variables analogously to (12) and adding the following *k* terms in the ascending order to the right of the matrix on the right hand side of (12)

$$\left(a_{iii},\ldots ,a_{iik},\ldots ,a_{i,k-1,k-1},a_{i,k-1,k},a_{ikk}\right)),\;i\in \;\left\{1,\ldots ,k\right\}$$

Functions quadr and cub create additional variables by introducing higher order terms, and are specifically designed for numerical continuous and binary variables and is not well-defined for non-numeric variables. Importantly, as previously stated in the Introduction, by allowing the predictive model to take a form of a polynomial, we enable it to unravel potential non-linear relationship between variables analogous to Taylor series approximation, which may consequently lead to a better prediction.

## Software implementation

The software package implementing the methods above is available under https://cran.r-project.org/web/packages/CARRoT/index.html with the main function: regr_ind delivering the analysis. The remaining ones mostly play auxiliary roles. For the linear mode specified as mode=’linear’ the function lsfit() is used to call linear regression. Multinomial and binary modes are specified as mode=’binary’ and mode=’multin’, respectively. We note that in both cases the function multinom() from the package *nnet* is used to run the corresponding regression.

We use R dataset swiss to illustrate the linear mode of regr_ind. We attempt to predict fertility (first variable in the dataset), which is a continuous variable using the remaining five variables. Sample size equals 47 which means that all models with up to 4 predictors are feasible for EPV = 10. The following line of code calls regr_ind with 1000 cross-validations.


>set.seed(657)}
>result<-regr_ind(vari=swiss[,2:6],outi=swiss[,1],crv=1000,mode='linear')


The partition into training and test set is determined by the parameter part with a default value 10 as in the above case, which means that the size of the training set is 1/10 of the size of the whole dataset. The number of hold-outs crv is set to a 1000. The first line of the output will be an array of six numbers, first two corresponding to the average absolute and relative errors attained on the test sets by the best performing models respectively. Furthermore, the second pair of numbers refers to the same types of errors reached by this model on the training sets. Comparing the difference between the average errors on the training and the test sets allows to evaluate degree of overfitting while training. Finally, the last two numbers are the average absolute and relative errors produced by the empirical prediction on the test set for each cross-validation. The idea behind this is to determine whether the prediction produced by the best feasible subset of the dependent variables is able to reach a higher predictive power than the most straightforward prediction, i.e., the sample mean.


[1] 6.05029812 0.08904263 5.42811143 0.07987293 9.72291238 0.15296233


The list object result consists of three lists: [[1]], [[2]] and [[3]], which correspond to the array of predictive powers printed out earlier and two lists of models exhibiting the lowest absolute and relative errors respectively, each model defined as a set of indices of the dependent variables constituting it. Note that the variable indices in the output are given with respect to the input dataset, swiss[,2:6] in this case, and not the entire dataset.


>result
[[1]]
[[1] 6.05029812 0.08904263 5.42811143 0.07987293 9.72291238 0.15296233}
[[2]]
[[2]][[1]]
[1] 1 3 4 5
[[3]]
[[3]][[1]]
[1] 1 3 4 5


In this particular case the same model exhibits the lowest average absolute and relative error. In order to display the names of the variables constituting the best model


>names(swiss[,2:6]) [result[[2]][[1]]]
[1] "Agriculture"    "Education"    "Catholic"    "Infant.Mortality"


By varying parameters such as the partition into training and test set (part), EPV rule (rule) and choosing the option of model assessment based on *R*^2^ statistic and global shrinkage factor (Rsq=TRUE) and others one can assess robustness of the model. For example, if on top of the default EPV = 10 we use Rsq=TRUE, the difference between the error on the training and test set, as well as the overall performance of the best model is only slightly better than in the default case Rsq=FALSE. The selected model is the same as in previous case.


>set.seed(657)
result<-regr_ind(swiss[,2:6],swiss[,1],1000,mode=’linear’,part=10,Rsq=T)
[1] 6.04956000 0.08900914 5.43180952 0.07990912 9.72291238 0.15296233
>names(swiss[,2:6])[result[[2]][[1]]]
[1] "Agriculture"    "Education"    "Catholic"    "Infant.Mortality"


In the above example we did not set the error margin for mean and variance estimators discussed in Section “Additional sample size criteria”, as the sample size of 47 subjects is rather small and low error margin will simply lead to all modes being infeasible. The output of the model will read


>set.seed(657)
result<-regr_ind(swiss[,2:6],swiss[,1],1000,\\
                                mode=’linear’,part=10,Rsq=T,marg=0.1)
[1] NA NA 9.72291238 0.15296233



NAs in the output refer to non-existence of any feasible model, performance on the training set is therefore omitted, and the last two values as before correspond to predictive power of the empirical prediction. On the other hand, if one chooses EPV = 20, the increase in the absolute error is smaller than in case EPV = 10 (8% vs 11%) whilst the absolute error itself increases by 20%. The selected model is now constituted by only two variables due to an EPV restriction.


>set.seed(657)
result<-regr_ind(swiss[,2:6],swiss[,1],1000,mode=’linear’,rule=20)
[1] 7.3065447 0.1061592 6.7645138 0.0981193 9.7229124 0.1529623
>names(swiss[,2:6])[result[[2]][[1>]]]
[1] "Education" "Catholic"


As a binary case example we use dataset Pima.tr from the package *MASS* [25], also known as “Diabetes in Pima Indian Women”. We use first seven variables of the dataset in order to predict whether a person has diabetes. Note that there are 68 subjects with diabetes overall.


>set.seed(100556)
regr_ind(Pima.tr[,1:7],Pima.tr[,8],crv=100,cutoff=0.5,\\
                                mode=’binary’,objfun=’acc’,part=5,maxw=5)
[1] 0.7635000 0.7841875 0.6602500
[[1]]
[1] 0.7635000 0.7841875 0.6602500
[[2]]
[[2]][[1]]
[1] 2 6 7
>names(Pima.tr[,1:7])[c(2,6,7)]
[1] "glu" "ped" "age"


In this case the best model is chosen via accuracy maximisation. This is indicated by objfun=’acc’, although could have been skipped since this is the default value for parameter objfun. The output consists of two lists. One is a list of accuracies, where the first and the second values correspond to the accuracy of the best model on the test and training set, respectively, and the third one corresponds to the accuracy of the empirical prediction. The second list is a list of the corresponding best models. Cut-off value for the deterministic prediction is specified via cutoff=0.5. The size of the sample is 200, with 68 events, which means that if one always predicts the absence of diabetes, accuracy of the prediction will be 0.66 (although for different partitions of the set into training and test sets the value may vary slightly, e.g. it reads 0.6603 in the example above). Input variable part indicates that the training set is 1/5 (20%) of the whole dataset. Note, that there are 68 adverse outcomes the probability of at least 60 of them occurring in the randomly chosen 160 subjects is around 0.026. This implies that, on average, out of 100 hold-outs, there will be 2–3 for which a model of weight 6 will be feasible, and for the remaining hold-outs it will not be assessed. In case such model performs well on those hold-outs, it will significantly bias the output since, as compared to smaller models, it was not cross-validated as many times. We tackle this problem by specifying parameter maxx=5 thereby restricting the maximum weight of the model to 5. Note that empirical prediction computes occurrence of the value 0, and hence if in a given dataset value 1 arises more frequently than 0 the output will be below 0.5. Similarly, one can change the objective function parameter to ’roc’ in order to identify a model with the highest average AUROC value. Note that in this case, empirical prediction is not reported.


>set.seed(100556)
regr_ind(Pima.tr[,1:7],Pima.tr[,8],100,cutoff=0.5,mode=’binary’,\\
                                           objfun=’roc’,part=5,maxw=5)
[1] 0.8223213 0.8504157
[[1]]
[1] 0.8223213 0.8504157
[[2]]
[[2]][[1]]
[1] 2 5 6 7
>names(Pima.tr[,1:7])[c(2,5,6,7)]
[1] "glu" "bmi" "ped" "age"


To illustrate multinomial mode of the package, we use the dataset bladder1 from the package *survival* [26]. We predict the status variable given the previous six variables, merging into one category events of type 2 and 3. The notation is as in the binary case


>bladder1[which(bladder1[,8]==3),8]=2
>set.seed(901)
>regr_ind(bladder1[,2:7],bladder1[,8],100,mode=’multin’)
[1] 0.8226667 0.8290152 0.6406667
[[1]]
[1] 0.8226667 0.8290152 0.6406667
[[2]]
[[2]][[1]]
[1] 4 5


Hold-out procedures within regr_ind can also be parallelised by means of parameter parallel=TRUE, which is set to FALSE by default, and specifying the number of cores. This is accomplished via the function foreach from the package *doParallel* and significantly enhances the computations as in the following example where “…” stands for the same values of parameters as in the case of no parallelisation.


>set.seed(100556)
>system.time(regr_ind(Pima.tr[,1:7],Pima.tr[,8],crv=100,\\
                            mode=’binary’,objfun=’roc’,part=5,maxw=5)))
[1] 0.8223213 0.8504157
   user  system elapsed
48.730   0.224  49.002
>system.time(regr_ind(vari=Pima.tr[,1:7],outi=Pima.tr[,8],\\
                                    … ,parallel=TRUE,cores=7))
[1] 0.8374064 0.8465505
   user  system elapsed
0.145   0.068  13.812


To take into account higher order interactions between variables, functions quadr and cub can be used. An example is the dataset rock from the package *datasets*, where the permeability of a rock sample is the dependent variable given the area of pores space, perimeter and shape.


> set.seed(111)
> regr_ind(rock[,1:3],rock[,4],1000,cutoff=0.5,mode=’linear’)
[1] 182.959213   2.173267 165.298121   1.887752 407.193904   9.600623
[[1]]
[1] 182.959213   2.173267 165.298121   1.887752 407.193904   9.600623
[[2]]
[[2]][[1]]
[1] 1 2
[[3]]
[[3]][[1]]
[1] 1 2 3

> set.seed(111)
> regr_ind(quadr(rock[,1:3]),rock[,4],1000,cutoff=0.5,mode=’linear’)
[1] 168.487046   1.363073 146.657772   1.153693 407.193904   9.600623
[[1]]
[1] 168.487046   1.363073 146.657772   1.153693 407.193904   9.600623
[[2]]
[[2]][[1]]
[1] 1 6 8
[[3]]
[[3]][[1]]
[1] 1 2 4 7


Note that in this case introduction of quadratic terms in the above case reduces the absolute error by around 8% and the relative one by more than 60%. Quadratic models also contain linear term ‘area’ (index 1) which was previously a part of the best linear model. The function find_int can be used to ‘decipher’ the index of the coefficient into the interaction between the original variables by using the size of the initial dataset:


>find_int(ind=6,N=3)
[1] 1 3


Therefore, index 6 given 3 variables is the interaction between the first one and the third one, which correspond to area and shape in this particular case.

## Results and discussion

### Predictive power

In this section, we provide examples of usage of *CARRoT* on a number publicly available datasets and compare its performance to other widely used variable selection techniques within the presented framework. In addition, we discuss the application of *CARRoT* in several published studies.

Firstly, we assess over a 100 datasets appropriate for predictive modelling available as part of different R-packages. These include 88, 32 and 8 instances used for continuous, binary and multinomial predictions, respectively. We choose the default *CARRoT* mode with EPV = 10 and 90% − 10% hold-out split for these datasets and report the predictive power quantified as absolute/relative error, accuracy/AUROC and accuracy only for each one of the respective package modes in S1 Table. The number of hold-outs is set to 1000 and the cut-off value in the binary mode equals 0.5, while neither *R*^2^ statistic nor global shrinkage factor are not taken into account.

Furthermore, we amend the above predictive models based solely on EPV rule with constraints on the *R*^2^ statistic and global shrinkage factor by setting Rsq parameter to TRUE, and investigate the subsequent change in the predictive power. Note that this method applies only to datasets where continuous and binary modes have been assessed.

Finally, using the same framework of multiple hold-outs we evaluate three types of models widely used in practice, namely variable selection based on statistically significant univariate regressions, stepwise forward selection and lasso regression.

For the first method, we build our comparison as follows: for each hold-out, we identify variables which are statistically significant (at 0.05 level) on the training set based on the results of univariate regressions, and compute the predictive power of the model which contains all such variables on the test set. Note that, for different partitions of data into training and test set, different variables may exhibit significance especially in the presence of data heterogeneity. No particular predictors are fixed in this case, and therefore, instead of computing the average predictive power of a particular model, we obtain the average predictive power achievable with this approach. In reality, this predictive power can be reached if and only if there is a certain set of predictors which are consistently statistically significant, independently of the nature of the hold-out split. Stepwise forward selection procedure is performed conceptually in the same way.

Similar holds for the case of lasso regression. We use hold-out splits in order to choose the penalty parameter λ which maximizes the predictive power. The set of penalty parameter values is therefore fixed across all hold-outs and the highest predictive power corresponds to the most optimal penalty parameter rather than to a set of predictors. Hence, as in the previous cases of significant variables and stepwise selection, we evaluate the approach as a whole, since a given parameter λ does not guarantee the same set of non-zero regression coefficients for each split of the data into training and test set.

For the first method, the p-value of *F*-statistic was computed by extracting the corresponding value from lm for linear and Anova from package *car* [27] for multinomial regression. For the stepwise selection, we employ function stepAIC from the package *MASS* [25], therefore using Akaike Information Criteria (AIC) to evaluate the models throughout each procedure. For lasso, we used glmnet from the corresponding package [28]. We make sure to use same exact hold-out splits for all types of models.

Note that in 76% and 69% of the cases predictions provided by *CARRoT* are strictly better than the ones obtained by the models built upon statistically significant explanatory variables and stepwise selection respectively, as presented in the Tables 2 and 3. On the other hand, lasso-based model exhibits strictly lower predictive power 50% of the time and in case the *CARRoT* model is constrained by *R*^2^-statistics the corresponding value goes down as low as 22% (see Tables 4 and 5). The remaining cases of the alternative models yielding higher or equal results largely fall into the following categories:

- Predictive power of *CARRoT* output is equal to the predictive power of the other model.
  Usually, this means that the output of *CARRoT* includes the same set of predictors selected by another model (and possibly other sets of predictors with the same exact predictive power). In particular, when comparing the model with additional *R*^2^ constraints to the simple EPV = 10 the latter result implies that the model satisfying EPV = 10 constraint and exhibiting the highest predictive power also satisfies the *R*^2^ constraint. Note that this occurs 67% of the time.
- Predictive power of *CARRoT* output is lower than the predictive power of another model since the sample size is too small for *CARRoT* to fit the latter model.
  This applies to lasso-based, significant predictors based and stepwise selection based models which are therefore likely to be overfitted.
  Examples of such datasets are immer, oats, mpg (see S1 Table). In each one of these datasets the sample size is low compared to the number of categories of the predictors and the lasso-based, significance-based and stepwise selection-based models violate the ‘rule of ten’.
- Predictive power of *CARRoT* output is lower than the predictive power of another model due to the fact that the set of selected predictors changes depending on the partition into the training and test set.
  The latter is again applicable to lasso-based, significance-based and stepwise selection-based models and indicates that the given dataset is rather heterogeneous, and that the model built on the basis of lasso regularisation or significant predictors is highly unreliable, and hence unsuitable for medical applications in particular.
  Examples of such datasets are urine, coop (see S1 Table) where the set of significant predictors, predictors selected during a stepwise procedure (in case of urine) or shrinked to 0 coefficients corresponding to the same penalty parameter λ changes depending on the partition into the training and test set).
  Similar situation arises when the *CARRoT* based model with additional *R*^2^ constraint exhibits higher predictive power than the model based on EPV alone: this indicates that, due to the *R*^2^ constraint, the best model was not feasible for a number of hold-out splits. This further indicates the presence of heterogeneity in the given dataset, which one can observe e.g. in uis
- Coefficient estimators of the lasso model, known to be biased towards zero lead to a higher predictive predictive power, even though the selected variables are the same as the ones picked by *CARRoT*, e.g. in environmental. In such cases other factors, such as overfitting of the model need to be assessed in order to conclude which coefficients are more reliable.

**Table 2:** Comparison of predictive power. Significance-based models. The table reports percentage of time one model yields better/worse predictive power than the other one.

		B better than S	B equals S	B worse than S	E better than S	E better than B
Linear	Absolute	85%	12%	3%	20%	3%
	Relative	71%	25%	4%	21%	0%
Binary	Accuracy	83%	13%	3%	16%	0%
	AUC	71%	23%	6%	-	-
Multin	Accuracy	44%	33%	22%	0%	0%
Overall		76%	19%	5%	19%	1%

‘S’ stands for the model constituted by all significant predictors, ‘B’ stands for the one provided by *CARRoT*, ‘E’ stands for the empirical model (where applicable). For example, in 85% of the cases *CARRoT* exhibits lower absolute error than the model based on statistically significant explanatory variables. All other values should be interpreted in a similar fashion.

**Table 3:** Comparison of predictive power. Stepwise selection-based models.

		B better than SF	B equals SF	B worse than SF	E better than SF	E better than B
Linear	Absolute	76%	8%	16%	15%	3%
	Relative	69%	23%	9%	15%	0%
Binary	Accuracy	60%	27%	13%	17%	0%
	AUC	57%	30%	13%	-	-
Multin	Accuracy	62.5%	12.5%	25%	0%	0%
Overall		69%	18%	13%	13%	1%

Notation as in Table 2. ‘SF’ stands for the stepwise forward selection model.

**Table 4:** Comparison of predictive power. Lasso-based models.

		B better than L	B equals L	B worse than L	E better than L	E better than B
Linear	Absolute	57%	10%	33%	8%	3%
	Relative	51%	28%	21%	1%	0%
Binary	Accuracy	48%	35%	16%	3%	0%
	AUC	35%	42%	23%	-	-
Multin	Accuracy	44%	11%	44%	0%	0%
Overall		50%	24%	26%	4%	1%

Notation as in Table 2. ‘L’ stands for the lasso-based regression model.

**Table 5:** Comparisons of predictive power. *R*^2^ based models.

		B better than R	B equals R	B worse than R	E better than R	E better than R
Linear	Absolute	30%	62%	8%	3%	1%
	Relative	20%	65%	15%	1%	0%
Binary	Accuracy	16%	78%	6%	0%	0%
	AUC	16%	78%	6%	-	-
Overall		22%	67%	10%	2%	1%

Notation as in Table 2. ‘R’ stands for the model with EPV = 10 and additional constraints based on *R*^2^-statistics.

Statistics on the results from S1 Table is summarised in Tables 2–6. Observe that, on average, absolute error exhibited by the models selected by *CARRoT* is 1.38 and 1.09 times smaller than the one obtained when using the models constituted of all significant predictors and predictors selected through a stepwise procedure respectively, whereas the corresponding relative errors are on average 1.66 and 1.53 times smaller. Accuracy and AUROC values produced by *CARRoT* are higher than the ones obtained by the two models. Indeed, the ratios read 1.03 and 1.02 for binary accuracy and binary AUROC modes, respectively. Lasso-based model performs mostly no better than *CARRoT* and analogous values from the Table 6 are mainly above 1. Note that all the outputs were rounded to the second decimal place prior to performance comparison.

**Table 6:** Comparisons of predictive power.

Linear							
S/B abs	S/B rel	SF/B abs	SF/B rel	L/B abs	L/B rel	R/B abs	R/B rel
1.38	1.66	1.09	1.53	1.04	1.09	1.66	1.06
Binary							
B/S acc	B/S AUC	B/SF acc	B/SF AUC	B/L acc	B/L AUC	B/R acc	B/R AUC
1.03	1.02	1.02	1.02	1.01	1.0	1.02	1.02
Multinomial							
B/S acc		B/SF acc		B/L acc		B/R acc	
1.0		1.0		0.96		1.74	

‘S’ stands for the model constituted by all significant predictors,‘SF’ stands for stepwise forward selection, ‘L’ stands for lasso-based method, ‘R’ stands for EPV = 10 with additional *R*^2^ constraints, ‘B’ stands for the one provided by *CARRoT* with EPV = 10, e.g., the value ‘S/B absolute’ of 1.28 means that, on average, the model constituted by all significant predictors leads to a 1.28 times higher absolute error than the one provided by *CARRoT*. In case of categorical variables ‘B/S accuracy’ value of 1.05 means that output of *CARRoT* yields 1.05 higher accuracy than the other model. All other values should be interpreted in a similar fashion.

We observe that in around 20%, 15% and 8% of the cases the absolute error of the significance-based, stepwise selection-based and lasso-based models is higher than the one provided by the sample mean, respectively. In contrast, in all cases considered, the *CARRoT* output both with and without additional *R*^2^-constraints, produces lower relative error than the empirical one whilst it yielded lower absolute error in at least 97% of the cases. We note that that for a fixed model of predictors, the numbers above might turn out to be even less favorable.

### Overfitting analysis

Frequently observed low difference in the predictive power between models described above suggests a need for a closer analysis of overfitting. We choose a representative sample of 44 datasets and assess each model. For this purpose, in addition to average predictive power on the test sets, we computed average predictive power on the respective training sets. We report the ratio between predictive power on the test set and the training set, with higher values of the latter indicating presence of overfitting.

Results of the overfitting analysis (S2 Table) are summarised in the Tables 7 and 8. We observe that the highest average overfitting is exhibited by the stepwise forward selection-based model and lasso-based model, reaching 14% and 10% respectively, whilst the lowest value is attained by the *CARRoT* with additional *R*^2^ constraint, although the latter outperforms the plain EPV-based only *CARRoT* by only a percent. In addition, we note that although stepwise selection based model outperforms the significance based one in terms of predictive power, its results are still worse than those of the EPV-based only model. Moreover, although in Table 8 the predictive power of EPV based only on *CARRoT* model is not always strictly higher than the one of the lasso-based model, the latter ones exhibit more overfitting, especially in case of binary outcomes where lasso-based models maximising AUC overfit more than *CARRoT* based ones in all cases (see Table 8). On the other hand, additional *R*^2^ constraint significantly reduces the overfitting, where positive values in the corresponding column of the upper part of Table 8 mainly account for the cases where the overfitting is equal between two models. We also assess the cases when EPV based only *CARRoT* yields both lower predictive power and higher degree of overfitting in the lower part of the Table 8. We report that EPV-based model performs worse than its significance-based, stepwise forward selection-based and lasso-based counterparts on average only 1%, 4% and 7% of the time, respectively. The highest value of 16% corresponds to EPV-based model with *R*^2^ constraint, which reflects mainly its superiority with respect to overfitting.

**Table 7:** Overfitting comparison. The table reports average absolute difference between overfitting defined in “Results and discussion” for different methods.

		Best	Significant	Stepwise	Lasso	R-squared
Linear	Absolute	6%	10%	15%	12%	5%
	Relative	6%	11%	13%	18%	5%
Binary	Accuracy	2%	3%	5%	4%	2%
	AUC	2%	4%	5%	4%	2%
Multin	Accuracy	1%	7%	10%	3%	-
Overall		5%	9%	14%	10%	4%

**Table 8:** Combined overfitting and predictive power comparison. The table summarises predictive power and overfitting.

Overfitting		B less than S	B less than SF	B less than L	B less than R
Linear	Absolute	59%	86%	63%	15%
	Relative	43%	76%	65%	13%
Binary	Accuracy	57%	86%	71%	0%
	AUC	47%	83%	100%	29%
Multin	Accuracy	50%	75%	63%	-
Overall		52%	77%	68%	14%
Predictive power		B better than S	B better than SF	B better than L	B better than R
Linear	Absolute	70%	64%	33%	30%
	Relative	65%	62%	37%	37%
Binary	Accuracy	86%	71%	57%	14%
	AUC	71%	50%	57%	57%
Multin	Accuracy	50%	75%	38%	-
Overall		67%	64%	38%	35%
Predictive power and overfitting		B worse than S	B worse than SF	B worse than L	B worse than R
Linear	Absolute	0%	1%	7%	11%
	Relative	0%	7%	11%	19%
Binary	Accuracy	0%	0%	0%	43%
	AUC	14%	14%	0%	0%
Multin	Accuracy	0%	0%	13%	-
Overall		1%	4%	7%	16%

Models notation as in Table 6. Upper table refers to percentage of time the first model exhibits lower overfitting than the second one. Middle table refers to percentage of time the first model exhibits higher predictive power than the second one. Lower table refers to the first model exhibiting both lower predictive power and higher overfitting than the second one.

We note that in more than 60% of the cases the EPV-based model output by *CARRoT* contains variables constituting a subset of those chosen by the lasso-based model. This, together with the increased presence of overfitting in the latter, suggests that in the presence of large numbers of variables when best subset selection constrained by EPV is computationally infeasible, a hierarchical procedure of model selection can be used. Namely, lasso as the first step of variable selection and subsequent best subset regression which returns unbiased regression coefficients estimators.

The model corresponding to Rsq=TRUE exhibits less overfitting, however majority of the time outputs exactly the same result as the EPV = 10 model suggesting that most of the models satisfying the latter constraint and exhibiting high predictive power also satisfy necessary conditions on the *R*^2^-statistics and global shrinkage factor. Moreover, the degree of overfitting on average is rather close for the two models as evidenced by the Table 7.

In order to assess how predictive power changes with the increase of EPV rules, we ran *CARRoT* with EPV values of 7, 10, 20, 30 and 40 predictors. In addition, we investigate the decrease in overfitting as EPV grows. The results are summarised in Tables 9 and 10. One can observe that for high values of EPV, such as 30 and 40, the overfitting is as low as 3% and 4%, respectively. At the same time, the average ratio of absolute error between EPV = 30 and EPV = 10, EPV = 40 and EPV = 10 is 1.09 and 1.4, respectively. On the other hand, the average values of overfitting and predictive power ratios for EPV = 20 are comparable to those ones provided by EPV = 10 with additional *R*^2^ constraints suggesting that the latter to an extent may be replaced by EPV = 20 being more user-friendly given its simplicity.

**Table 9:** Overfitting comparison for different EPVs. The table reports the average absolute difference between overfitting defined in Results and discussion for different EPV rules.

EPV		7	10	20	30	40
Linear	Absolute	7%	6%	5%	3%	2%
	Relative	6%	6%	4%	3%	2%
Binary	Accuracy	3%	2%	1%	2%	1%
	AUC	3%	2%	1%	0.5%	1%
Multin	Accuracy	2%	1%	1%	2%	2%
Overall		5%	5%	4%	3%	2%

**Table 10:** Predictive power for different EPVs and *R*^2^. The average ratios between predictive power of models with different EPVs as in Table 6.

EPV ratio		7/10	20/10	30/10	40/10	*R*^2^/10
Linear	Absolute	0.99	1.04	1.09	1.4	1.06
	Relative	0.99	1.03	1.05	1.08	1.05
EPV ratio		10/7	10/20	10/20	10/20	10/*R*^2^
Binary	Accuracy	0.99	1.01	1.03	1.04	1.0
	AUC	0.98	1.01	1.04	1.07	1.0
Multin	Accuracy	1.01	1.02	1.01	1.02	-
Overall		0.99	1.03	1.06	1.21	1.04

E.g. 7/10 means average ratio between predictive power provided by EPV = 7 and EPV = 10. The last column corresponds to ratio of predictive power of EPV = 10 model with additional *R*^2^ statistics constraint and EPV = 10 model only.

We provide several examples when including second-order interactions between independent variables significantly boosts the predictive power of the model and present our findings in the Table 11. Note that we used same exact partitions of the datasets into training and test ones for linear and quadratic models, in order to see whether inclusion of quadratic terms yields higher performance characteristics. Indeed, in all cases considered, the predictive power increases by at least 5%, whilst there are cases where absolute error shrinks almost twice. Moreover, we report the degree of overfitting for both linear and quadratic cases. We find that in more than 50% of the datasets, the latter did not grow despite the increased complexity of the predictive model and significant improvement in predictive power. The latter suggests the presence of non-linearity in the relationship between the outcome and explanatory variables.

**Table 11:** Best performance based on linear and quadratic model.

Dataset	M	Out	Linear	OF	Quadratic	OF	Empirical	N
kidrecurr (KMsurv)	l	time2	92.71/3.27	0.04/0.02	85.98/1.64	0.05/0.04	100.57/3.74	5
hodg (KMsurv)	l	time	381.54/6.42	0.10/0.08	354.33/3.41	0.10/0.07	445.68/11.16	4
myeloid (survival)	l	rltime	137.75/0.59	0.03/0.01	128.70/0.53	0.01/0.02	193.89/0.91	3
snails (MASS)	l	Temp	1.54/*NA*	0.06/*NA*	1.23/*NA*	0.09/*NA*	2.99/*NA*	3
environmental (lattice)	l	ozone	16.74/0.70	0.03/0.03	14.02/0.47	0.06/0.05	27.06/1.59	3
Phenobarb (nlme)	l	dose	4.77/0.73	0.01/<0.01	3.92/0.62	0.01/0.01	5.16/1.0	3(4)
Wheat (nlme)	l	DryMatter	2.06/0.29	0.07/0.04	1.10/0.16	0.10/0.07	3.22/0.50	3
Wheat2 (nlme)	l	yield	4.54/0.41	0.01/0.05	4.07/0.33	0.03/0.01	5.42/0.57	4
rock (datasets)	l	perm	183.97/2.15	0.12/0.13	168.51/1.32	0.14/0.13	407.50/9.24	3
channing (boot)	b	cens	0.64/0.67	0.01/0.02	0.71/0.79	0.01/0.01	0.62/−	3(4)
nodal (boot)	b	r	0.74/0.69	0.01/0.01	0.78/0.73	0.01/0.01	0.62/−	5
bladder (survival)	b	event	0.72/0.77	0.02/0.01	0.77/0.81	<0.01/0.02	0.67/−	4

Column ‘Dataset’ corresponds to the R dataset analyzed, while the package it originates from is provided in the brackets. Column ‘M’ corresponds to the value of mode parameter of regr_ind or regr_whole. ‘Out’ is the name of the dependent variable from the corresponding dataset. Columns ’linear’, ‘Quadratic’ and ‘Empirical’ correspond to the values of predictive power attained by the best linear, best quadratic and empirical (were applicable) models, respectively. For the mode linear, the value in these columns stands for the absolute error/relative error, for the mode binary it corresponds to accuracy/AUROC, while it simply represents accuracy for multin mode. Column ‘Num’ stands for the overall number of numeric predictive variables in the linear model, while the number of all variables, including non-numerical ones (if any), is provided in the brackets. Columns ‘OF’ contain the overfitting measure for the respective model.

*CARRoT* was also used for building predictive models in a number of recently published studies. The Birmingham score for assessing patients with lower gastrointestinal bleeding was created based on the predictive model delivered by *CARRoT*, [29]. On top of outperforming the existing scores it is also simpler and easier to implement in practice. *CARRoT*-based models exhibited high predictive power when assessing the binary and multinomial patient outcomes following mechanical thrombectomy in stroke patients [30]. In patients with a functional stroke condition, *CARRoT* predicted reduction in the MRS and NIHSS scores following hypnotherapy sessions [31]. In [32], *CARRoT* was used to predict the cancer cell type based on the available explanatory variables and their second order interactions and yielded the accuracy of 92% with a quadratic model improving the linear one by 12%. In the metabolomics project, the package exhibited up to 0.91 AUROC when discriminating the types of renal tumours [33]. In the recent study in economics [34] *CARRoT* was used to build quadratic models in order to predict the presence of stochastic resonance based on certain parameters and reached AUROC of up to 0.9.

We intentionally made *CARRoT* compute and output a limited number of values and at times deliberately avoided usage of already existing R tools with the idea of saving computational time. For instance, combination of generic R functions predict() and glm() performed significantly slower than *CARRoT* function get_predictions(). On the other hand, due to a relatively simple structure of the main functions, it is straightforward to build-in new features and create additional objective functions if needed.

Moreover, if the set of independent variables is too large for performing exhaustive search, the concept of maximising predictive power over the test sets provided by *CARRoT* can be adapted to other types of variable selection, such as stepwise regression or backward elimination. All one needs to do is to restrict the number of variables in the model from below or above, fix certain variable in the model and then run *CARRoT* multiple times until the increment (decrease) in the predictive power is small (large) enough. This type of procedure was implemented in [35], where a modified version of the package was used in order to predict locations of the DNA replication origins and to take specific features of the dataset with no explicit negative instances into account.

There exists a number of R packages enabling exhaustive search for the best regression model, mentioned in the Introduction. Some of these packages have features intersecting with those of *CARRoT*, e.g., the feature restricting the number of variables in a model is in some cases equivalent to EPV rules. However, in *CARRoT*, the feature of computing maximum number of variables based on the sample size given a certain EPV rule is automatically built-in. Moreover, in cases when the set of explanatory variables is a mixture of categorical and continuous ones, the concept of restricting the number of variables from above is not necessarily equivalent to applying an EPV rule. Another important difference is that *CARRoT* has a built-in cross-validation procedure. Existing R packages for best subset regression mainly utilise Information Criteria or R-squared statistics for model ranking, whilst *CARRoT* works directly with the predictive power of each model as computed over the test sets. Large number of internal cross-validations ensures the approach provides robust results. Package *CARRoT* utilises linear regression models with Gaussian noise and multinomial logistic regressions. Note that the latter ones are not available as part of glm function, used in glmulti and bestglm, for dependent variables with more than two categories. In other words, *CARRoT* is a simple stand alone tool simultaneously combining the model selection, validation and controlling for overfitting.

## Conclusions

We developed an R-package for predictive modelling *CARRoT* by integrating sample size calculation rules, expert knowledge, cross-validation, best subset regression in one simple stand-alone tool. The package provides predictions for both continuous and categorical dependent variables.

We compared *CARRoT*’s performance to other widely used interpretable methods of model selection, such as Lasso regression, regression based on significant predictors, stepwise forward selection based on AIC, as well as different methods within *CARRoT* itself both in terms of exhibited predictive power and degree of overfitting. Best subset regression restricted by EPV = 10 shown very good results both in terms of predictive power and overfitting. With an additional constraint on the *R*^2^-statistic, overfitting decreases even further whilst predictive power does not encounter a significant drop. *CARRoT* drastically outperforms the models based on significant predictors alone and on predictors selected by stepwise forward selection procedure (over 75% and 69% of the time respectively and yields strictly higher predictive power than lasso regression 50% of the time. Moreover, *CARRoT* yields twice lower overfitting than the latter. We therefore conclude that predictive models produced by *CARRoT* are both robust and reliable. We believe that inclusion of expert knowledge (if available for particular dataset of interest) will further emphasise the difference in predictive power between the *CARRoT* output and the traditional models, as will including pairwise and/or three-way interactions between variables. The latter is a proxy for multidimensional Taylor series expansion, and therefore *CARRoT* has a strong potential to identify the non-linear relations (if present) between independent and dependent variables.

*CARRoT* allows users to select the best regression model based on a number of objective functions computed over the test sets depending on the outcome type. Other types of objective functions can be easily implemented in the future if necessary.

This package is a user-friendly universal tool designed to be able to deal with different data types, and so far it proved to be useful in Medical Statistics [29, 31–33]. It can also be used as a straightforward first step of data exploration before moving to more complex analysis techniques, if necessary.

## Supporting information

S1 Table Detailed breakdown of performance comparison of CARRoT output and the other models. Performance in terms of absolute/relative error, accuracy/AUROC and accuracy only (for continuous, binary and multinomial outcomes respectively) of different prediction methods on 100 datasets available in R using the default 90%/10% training/validation split. The methods used are *CARRoT* with EPV = 10, model, based on significant predictors only, lasso-based model, *CARRoT* with EPV = 10 and additional *R*^2^ constraint.
(CSV)

S2 Table Detailed breakdown of overfitting comparison of *CARRoT* output and the other models. Overfitting in terms of absolute/relative error, accuracy/AUROC and accuracy only (for continuous, binary and multinomial outcomes respectively) computed both on training and test sets of different prediction methods on 43 datasets available in R using the default 90%/10% training/validation split. The methods used are *CARRoT* with EPV = 10, model, based on significant predictors only, lasso-based model, *CARRoT* with EPV = 10 and additional *R*^2^ constraint.
(CSV)
